# Identification of the c.829_832delAATA Deletion Variants in the *BRCA1* Gene Associated with Hereditary Breast/Ovarian Cancer ˗ Case Report

**DOI:** 10.7150/jgen.68220

**Published:** 2022-02-14

**Authors:** Malgorzata Ostrowska, Karolina Olszewska-Bozek, Justyna Podlodowska, Jadwiga Sierocinska-Sawa, Jacek Wojcierowski

**Affiliations:** 1 Department of Biotechnology, Microbiology and Human Nutrition, University of Life Sciences in Lublin, 8 Skromna St., 20-704 Lublin, Poland; 2 Laboratory of Genetic Investigations, B. Dobrzanskiego 7 St., 20-262 Lublin; 3 Center of Oncology of the Lublin Region St. Jana z Dukli, 7 K. Jaczewskiego St., 20-090 Lublin; 4 Pathomorphology Department of Clinical Hospital I, I Independent Clinical Hospital, Stanislawa Staszica 16 St., 20-081 Lublin

**Keywords:** *BRCA1*, * BRCA2*, ovarian/breast cancer, NGS, PARP inhibitors

## Abstract

Determination of the *BRCA1/BRCA2* mutation status in patients with breast and/or ovarian cancer is commonly performed using various molecular techniques. The use of targeted PCR-based tests only may not be sufficient, as not all possible variants are investigated. In the present study, we used next-generation sequencing (NGS) techniques to identify novel pathogenic variants in *BRCA1* and *BRCA2*.

In this study, material (blood and FFPE) collected from a 67-year-old patient with ovarian cancer was used. The presence of hereditary mutations characteristic for the Polish population was examined using Sanger sequencing. *BRCA1* and *BRCA2* gene exons were amplified using the Devyser BRCA kit and sequenced on the Miniseq. No germline mutations characteristic for the Polish population were detected. However, 12 single nucleotide variants and 2 indels were identified. We found a new deleterious mutation of gene *BRCA1* (c.829_832delAATA). To our knowledge, this mutation has not been reported yet in the Polish population and elsewhere. The use of the NGS technique increases the possibility of detecting mutational changes in patients with ovarian and/or breast cancer. Quick determination of pathogenic variants is important to facilitate specific therapy, in addition to the identification of familial predisposition to cancer.

## Introduction

Ovarian cancer was the eighth most common neoplasm in women and the 18^th^ of all malignancies in the world in 2018 1. In 2018, there were nearly 295,400 new cases of ovarian cancer in the world and 5,077 in Poland 2. Approximately, 15% of ovarian cancers are caused by germline *BRCA1/2* (Breast cancer type 1 susceptibility protein and Breast cancer type 2 susceptibility protein) genes mutations 3, while 3% to 9% are related to pathogenic somatic variants 4.

The *BRCA1* gene (OMIM#13705) is located on chromosome 17q12. It encodes a multifunctional protein involved in homologous recombination DNA repair 5, G2 cell cycle checkpoint regulation 6, cell survival, and chromosome stability 7. The RING finger domain binds Bard1.p and Ola1.p forming an E3 ubiquitin ligase complex 8.

Germline mutations of the *BRCA1* gene have been detected in the majority of familial breast and ovarian cancers. Additionally, 30% to 40% of sporadic cases are associated with altered expression of *BRCA1* 9. In the Polish population, the most commonly studied mutations in the *BRCA1* gene include: 5382insC (c.5266dupC; p.Gln1756Profs), 300T>G (c.181T>G; p.Cys61Gly), 185delAG (c.68_69delAG; p.Glu23Valfs), and 4153delA (c.4035delA; p.Glu1346Lysfs) 10, 11.

*BRCA1* mutations associated with breast cancers are usually basal-like and triple-negative without *HER2* gene amplification 12. The risk of breast cancer for *BRCA1* and* BRCA2* germline mutations among the carriers under 80 years of age is approximately 72% and 69%, respectively. Similarly, the risk of serous ovarian cancer is 44% for *BRCA1* and 17% for *BRCA2* 13, 14. Homozygous nonfunctional mutations of *BRCA1* usually result in embryo lethality. The biallelic compound heterozygous variant of *BRCA1* containing c.594_597del4 and c.5095C>T is viable but exhibits susceptibility to microsomia, dysmorphia, mild intellectual disability, crosslinking agents, and breast cancer 15.

Here, we present a case report of serous ovarian cancer in a 67-year-old female patient and a novel germline mutation that has not been yet described either in Polish patients or in any other population.

## Materials and methods

### Study subjects

The examined material included blood cells (BC) and formalin-fixed paraffin-embedded tissue (FFPE) collected from a proband during standard laboratory procedures. The histological diagnosis of ovarian serous carcinoma and the tumor tissue was evaluated by a pathologist. Blood samples were collected from the proband's daughter, who had breast cancer in 2017, and from the proband's healthy son.

The patient, a 67-year-old female, underwent right-sided mastectomy for breast cancer (NST, G2, T2N0M0) in 2011 and complementary chemotherapy (FEC) in 2012. Furthermore, she suffered from hypogastric pain in July 2017, while ascites and tumors of the left and right adnexa were detected in September of the same year. The level of CA125 was 14749 U/ml. The use of standard tests did not reveal the presence of founder mutations in *BRCA1/BRCA2* characteristic of the Polish population.

The patient was treated with chemotherapy: 6 courses of Carboplatin AUC5 and Paclitaxel, which brought significant regression of the symptoms. In April 2018, the patient underwent radical surgery i.e. bilateral oophorectomy and hysterectomy. Diagnosis: G2 ovarian serous cancer with metastases to the abdomen. In November 2018, disease progression was recorded. In January 2019, the patient was reoperated on, and second-line chemotherapy was implemented: 6 courses of Carboplatin and Paclitaxel with complete response in imaging and marker tests. *BRCA1/2* mutation tests in archival material were ordered. A new, probably pathogenic* BRCA1* gene variation was found using the NGS technology. The patient was qualified for maintenance therapy with Olaparib (800 mg daily).

Approval for this study was obtained from the Bioethical Commission in Lublin (approval number 10/2020/KB/VIII). All procedures were performed in accordance with the Declaration of Helsinki (ethical principles for medical research involving human subjects).

### DNA Extraction and Genotyping

Genomic DNA was extracted from the whole blood using a commercial kit NucleoSpin Dx Blood (Machery-Nagel, Duren, Germany) according to the manufacturer's instructions. The quantity and quality of DNA were determined with the use of a BioPhotometer (Eppendorf, Hamburg, Germany). Three mutations of the *BRCA1* gene: 5382insC (c.5266dupC), 300T>G (c.181T>G), and 4153delA (c.4035delA) were amplified by PCR using primers and conditions described by 16,17. For other mutations: 185delAG (c.68_69delAG; p.Glu23Valfs), 3819delGTAAA (c.3700_3704delGTAAA; (p.Val1234Glnfs), 3875delGTCT (c.3756_3759delGTCT; p.Ser1253Argfs), 3896delT (c.3779delT; p.Leu1260Tyrfs), 4160delAG (c.4041_4042delAG, p.Gly1348Asnfs), and 4184delTCAA (c.4065_4068delTCAA; p.Asn1355Lysfs), primers were designed, and reaction conditions were experimentally determined (Table S1).

Primers were designed using the freely available Software Primer 18. The Taq PCR Master Mix kit (EURx, Gdansk, Poland) was used to prepare the PCR reaction (with 40 - 60 ng DNA) in a SimplyAmp^TM^ thermal cycler (Applied Biosystems, Thermo Fisher Scientific). The PCR products were enzymatically purified using an ExoBap kit (EURx, Gdansk, Poland). The sequential PCR was then prepared using a BigDye Terminator v3.1 Cycle Sequencing Kit (Applied Biosystems). In the next step, the terminators were removed after PCR using ExTerminator (A&A Biotechnology, Gdynia, Poland). After the purification, all samples were placed on a 96-well plate and sequenced using a 3100 Capillary Sequencer (Applied Biosystems, Thermo Fisher Scientific). Next, the results were pre-analyzed by ABI3100 Data Collection Software. Then, the data files of the samples were checked by FinchTV1.4 (Geospiza) and compared with reference sequences *BRCA1*: NM_007294.3.

### Multiplex Ligation-Dependent Probe Amplification (MLPA) Analysis

*BRCA1/2* genomic arrangements were searched with the MLPA method 19 according to the manufacturer's instructions (MRC-Holland, Amsterdam, the Netherlands) using P087 SALSA and P090 SALSA MPLA kits. The products of MPLA reactions were diluted 10 times in Hi-Di^TM^ Formamide (Applied Biosystems), and GeneScan^TM^ 500 ROX^TM^ dye Size Standard (Applied Biosystems) was added to each sample (as an internal lane size standard). It facilitated automated data analysis. Moreover, it was essential for the achievement of high run-to-run precision in sizing DNA fragments by electrophoresis. The samples were size-separated by capillary electrophoresis (POP7 polymer, ABI PRISM 3100, Applied Biosystems). The electropherograms were analyzed by GeneMarker software (version 2.2.0, SoftGenetics, State College, PA, USA).

### NGS Sequencing

The number of cancer cells (evaluated by the pathologist) was approximately 100% in the FFPE sample. DNA was extracted using GeneRead DNA FFPE (QIAGEN, Hilden, Germany). The DNA concentration was determined using a Qubit I fluorometer and a dsDNA High Sensitivity Assay Kit (Invitrogen^TM^, ThermoFisher Scientific).

The DNA was diluted to a concentration of 10 ng/µl. The libraries were prepared using a Devyser *BRCA* kit (Hagersten, Sweden) according to the manufacturer's instructions. Complete sequence determination of all coding exons and all adjacent exon-intron boundaries (minimum 20 bp proximal to 5' end and 10 bp distal to 3' end of each exon) were covered. First, amplicon libraries were generated in one multiplex PCR reaction (PCR1). Next, index addition to PCR1 was performed in PCR2. The sample libraries obtained in PCR2 were pooled and purified in a single tube. The library concentration was quantified using a Qubit I fluorometer and a dsDNA High-Sensitivity Assay Kit (Invitrogen^TM^, Thermo Fisher Scientific). The purified library pool was diluted to a 0.4 ng/ml concentration and denatured.

Sequencing was performed on MiniSeq (Illumina, San Diego, USA) using Mid Output Reagent Cartridge 300 cycles (Illumina, San Diego, USA). The Devyser *BRCA* libraries were sequenced in a paired-end mode (2 x 151 bp). The results of sequencing data files (FASTQ) were reviewed using Amplicon Suite (Smartseq s.r.l).

### Data Analysis

All mutations were reported following the Human Genome Variation Society (HGVS) guidelines (http://varnomen.hgvs.org/) based on the coding sequences: NM_007294.3 for *BRCA1* and NM_000059.3 for *BRCA2*. For the detection of sequence variants of somatic origin, we ensured that each amplicon had at least 1,000 coverage for detection of variant allele frequency (VAF) down to 5%. Mutations were classified according to clinical variants (https://www.ncbi.nlm.nih.gov/clinvar/), mutations in the COSMIC database (https://cancer.sanger.ac.uk/cancergenome/projects/cosmic/), variants of unknown significance, literature search, and *in silico* analyses using Varsome (https://varsome.com/). The presence of *BRCA1/2* genes mutations was confirmed by Sanger sequencing (Table S1).

## Results

**Figure 1:**
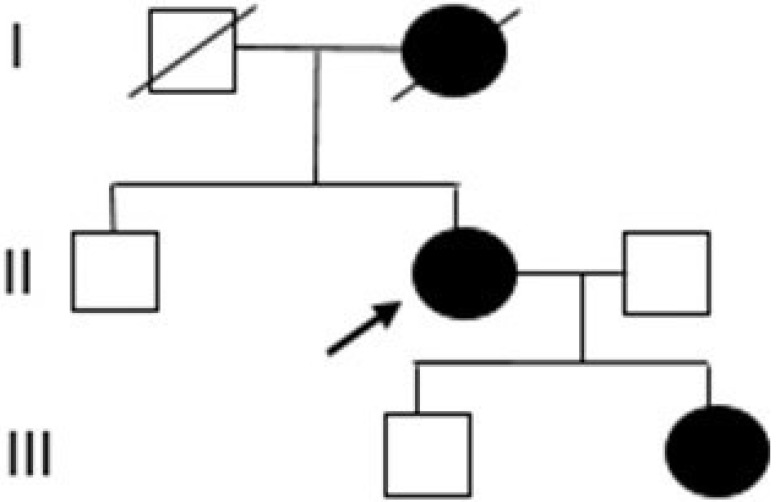
** Pedigree of the proband's family.** (I) mother with breast cancer, (II) proband with breast and ovarian cancer, and (III) daughter with breast cancer.

The proband was referred to genetic counseling since her mother was diagnosed with breast cancer (Figure 1).

Nine mutations in *BRCA1* were genotyped, i.e., c.5266dupC (p.Gln1756Profs), c.181T>G (p.Cys61Gly), c.68_69delAG (p.Glu23Valfs), c.4035delA (p.Glu1346Lysfs), c.3700_3704delGTAAA (p.Val1234Glnfs), c.3756_3759delGTCT (p.Ser1253Argfs), c.3779delT (p.Leu1260Tyrfs), c.4041_4042delAG (p.Gly1348Asnfs), and c.4065_4068delTCAA (p.Asn1355Lysfs), and were not detected in the samples from the proband.

MLPA analysis was used to test the presence of large genomic rearrangements (LGRs) in *BRCA1* and *BRCA2*. No deletions or duplications of genomic DNA fragments, i.e., copy number variations (CNV), were detected in the patient and patient's daughter samples (Figures S1A, S1B, S1C, S1D).

The data coverage for the FFPE sample showed a mean amplicon reading depth per sample ranging from 9813 to 2690 for *BRCA1* and from 17980 to 3237 for *BRCA2*. Seven single nucleotide variants (SNVs) and one deletion were identified in the *BRCA1* gene by NGS, whereas five SNVs and one deletion were detected in *BRCA2* (Table 1).

All detected SNVs of *BRCA1* were reported in ClinVar as benign (class 1). In this case, one probably pathogenic variant in *BRCA1* was identified: c.829-832delAATA, p.Asn277Leufs*20, NM_007294. This genetic variant is a frame-shift mutation resulting in a premature stop codon and protein truncation. This mutation is located in exon 10 of 23 (159 - 162 of 3426 codings, NMD).

**Figure 2:**
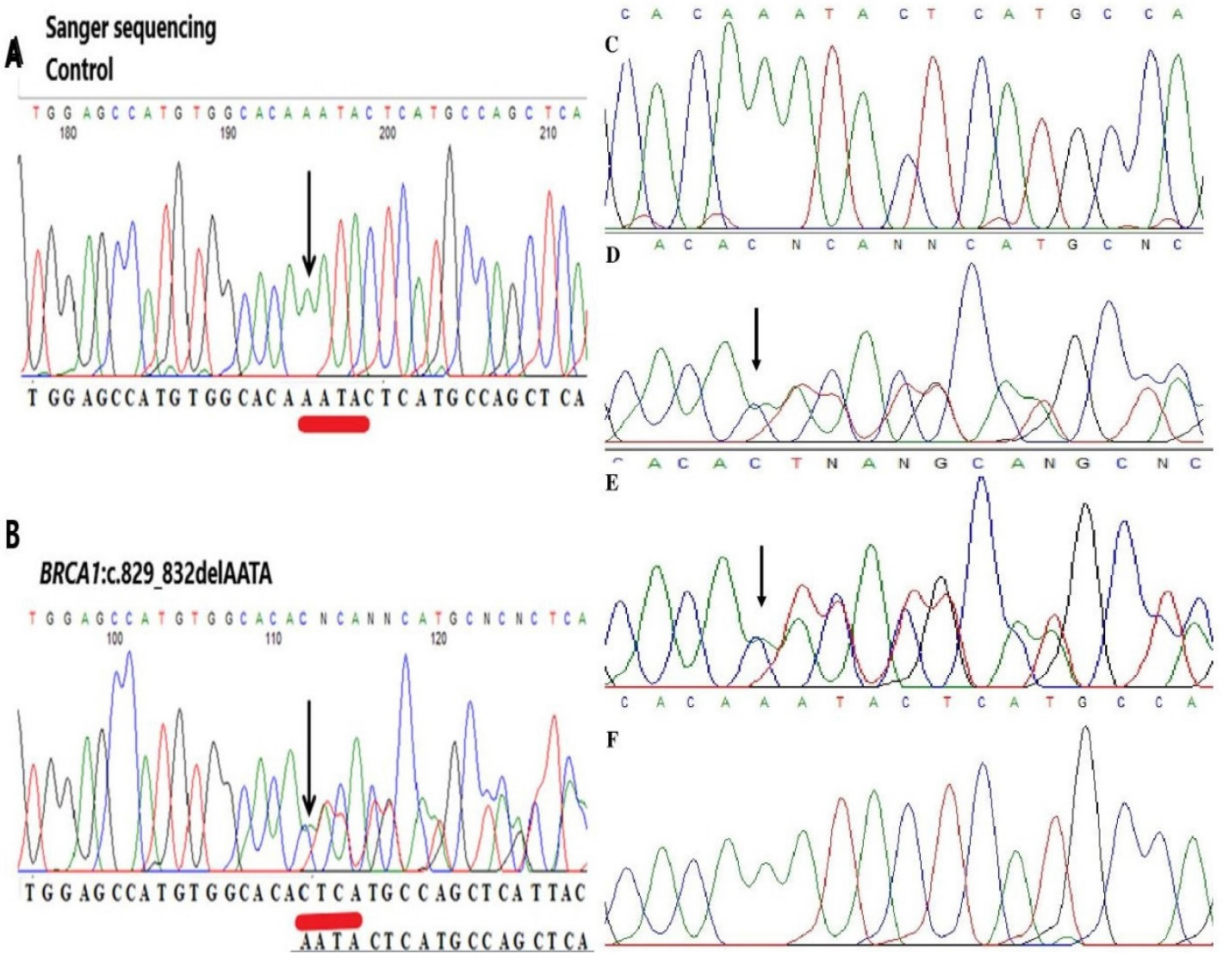
** Representative image of validation of NGS data by Sanger sequencing.** (A, C) Sequencing analysis of genomic DNA from the control. (B, D) Sequencing analysis of genomic DNA from the proband carrying the c.829_832delAATA mutation in the *BRCA1* gene. (E) Sequencing analysis of genomic DNA from the daughter carrying the c.829_832delAATA mutation in the *BRCA1* gene. (F) Sequencing analysis of genomic DNA from the son in the *BRCA1* gene. The arrows indicate the position of the mutated nucleotides.

To our knowledge, this mutation has not been reported yet in the Polish population and elsewhere. Additionally, this variant was fully confirmed by Sanger sequencing; therefore, the estimated specificity was 100% (Figure 2). A new variant was detected in the proband and the proband's daughter. This change was not detected in the son's samples.

Five detected *BRCA2* SNVs were reported as benign (class 1). One variant c.68-7delT was reported as a conflicting interpretation of pathogenicity (class 3) (Table 1).

## Discussion

Determination of the status of mutations in the *BRCA1/2* genes is important for the identification of a predisposition to familial cancer and for the selection of proper therapy. Hereditary mutations in the *BRCA1* and *BRCA2* genes are risk factors for ovarian cancer. Especially drugs based on PARP inhibitors and platinum can be used in patients with such mutations. New techniques can be used for the analysis of not only germline mutations but also somatic gene variants.

In *BRCA1*, one deletion c.829_832delAATA was detected by NGS. According to the American College of Medical Genetics and Genomics (ACMG) guidelines 20, 21, this variant may be classified as pathogenic, fulfilling the PVS1 criteria (pathogenic, very strong), PM2 (pathogenic moderate), or PP3 (supporting), and associated with breast and ovarian cancer. The pathogenic PVS1 criterion means a null variant (frame-shift), which is a known mechanism of disease. The *BRCA1* gene has about 2,908 known pathogenic variants (which is greater than the minimum of 0.7) associated with breast-ovarian cancer, familial, susceptibility to, 1, pancreatic cancer, susceptibility to, 4 and Fanconi anemia, complementation group S 20, 21.

The PM2 criterion means absent in controls (or at an extremely low frequency if recessive) in the Exome Sequencing Project, 1000 Genomes Project, or Exome Aggregation Consortium. Variants not found in GenomAD exomes coverage = 62.4 and not found in GnomAD genomes (good GnomAD genomes coverage = 32.2). PP3 pathogenic verdict computational was defined on 1 prognosis (GERP) vs. no benign. The germline *BRCA1* mutation c.829_832delAATA was confirmed as well (Figure 1).

The NGS analysis of *BRCA2* in the FFPE sample showed five SNV-type variations and one deletion. The c.68_7delT variant was not precisely qualified according to the ClinVar database. Two of the four annotations describe it as a variant of uncertain significance (VUS) (class 3) according to the ACMG guidelines 20, and the other two as benign (class 1) variants 21. The https://varsome.com/ database based on the American College of Medical Genetics and Genomics Classification indicates this variant as a change of unknown significance (class 3**)** 21.

In this case, no mutation previously described as a Polish-founder mutation was identified. The NGS-based testing allowed optimization of the detection of new variants of the *BRCA1/2* genes.

The use of PARP inhibitors in targeted therapy is beneficial for patients with *BRCA1/2* mutations 22, 23, 24. Parp1 plays a key role in the repair of single-strand DNA breaks 25. Recent studies indicate that NGS is increasingly being used in routine testing by laboratories. The use of this technology brings benefits to patients in terms of analysis time. NGS is recognized as an efficient method in the detection of both inheritable and acquired mutations using DNA from FFPE 26.

## Conclusions

In our work, we demonstrated that the use of standard procedures may not be sufficient in the diagnosis of hereditary and somatic *BRCA1/BRCA2* mutations. We have shown that NGS with a commercial kit for detection of *BRCA1/2* mutants from FFPE tumor tissue is fully efficient. Quick determination of pathogenic variants is important to facilitate specific therapy, in addition to the identification of familial predispositions to cancer. Discovering new variants that are inherited and that can cause familial cancers will facilitate monitoring other family members. Such an approach may help in the faster detection of undesirable changes that may cause cancer.

## Supplementary Material

Supplementary figures and table.

## Figures and Tables

**Table 1:** *BRCA1* and *BRCA2* variants detected by NGS

c.DNA	Protein	Type	RS number	Classification	% reads
*BRCA1*					
c.2612C>T	p.Pro871Leu	SNV	rs799917	benign [class 1]	5.96
c.4308T>C	p.Ser1436=	SNV	rs1060915	benign [class 1]	5.58
c.3113A>G	p.Glu1038Gly	SNV	rs16941	benign [class 1]	5.85
c.2311T>C	p.Leu771=	SNV	rs16940	benign [class 1]	5.94
c.2082C>T	p.Ser694=	SNV	rs1799949	benign [class 1]	6.23
c.3548A>G	p.Lys1183Arg	SNV	rs16942	benign [class 1]	6.63
c.4837A>G	p.Ser1613Gly	SNV	rs1799966	benign [class 1]	5.69
c.829_832delAATA	p.Asn277Leufs20	del		Pathogenic [class 5]	94.75
*BRCA2*					
c.68-7delT		del	rs276174878	Uncertain significance [class 3]	16.58
c.6513G>C	p.Val2171=	SNV	rs206076	benign [class 1]	99.29
c.4563A>G	p.Leu1521=	SNV	rs206075	benign [class 1]	99.24
c.7806-14T>C		SNV	rs9534262	benign [class 1]	50.87
c.7397T>C	p.Val2466Ala	SNV	rs169547	benign [class 1]	99.4
c.3807T>C	p.Val1269=	SNV	rs543304	benign [class 1]	50.93

## Abbreviations

ACMG: American College of Medical Genetics and Genomics
BC: Blood Cells
BRCA1: Breast Cancer 1, Early Onset
BRCA2: Breast Cancer 2, Early Onset
CNV: Copy Number Variants
FFPE: Formalin-Fixed Paraffin-Embedded
HGVS: Human Genome Variation Society
LGR: Large Genetics Rearrangement
MLPA: Multiplex Ligation-Dependent Probe Amplification
NGS: Next Generation Sequencing
PARP: Poly (Adenosine Diphosphate)-Ribose Polymerase
SNV: Single Nucleotide Variant
VAF: Variant Allele Frequency
VUS: Variant as of Uncertain Significance
