# Supplementary material for: Identification of the c.829_832delAATA Deletion Variants in the BRCA1 Gene Associated with Hereditary Breast/Ovarian Cancer ˗ Case Report
Source: J Genomics. 2022 Feb 14;10:33–8. doi: 10.7150/jgen.68220 (PMC8922303; doi:10.7150/jgen.68220)
Supplement: Supplementary file 1 — Supplementary figures and table. [file jgenv10p0033s1.pdf]

Supplementary materials

Supplementary figures S1A, S1B, S1C, S1D

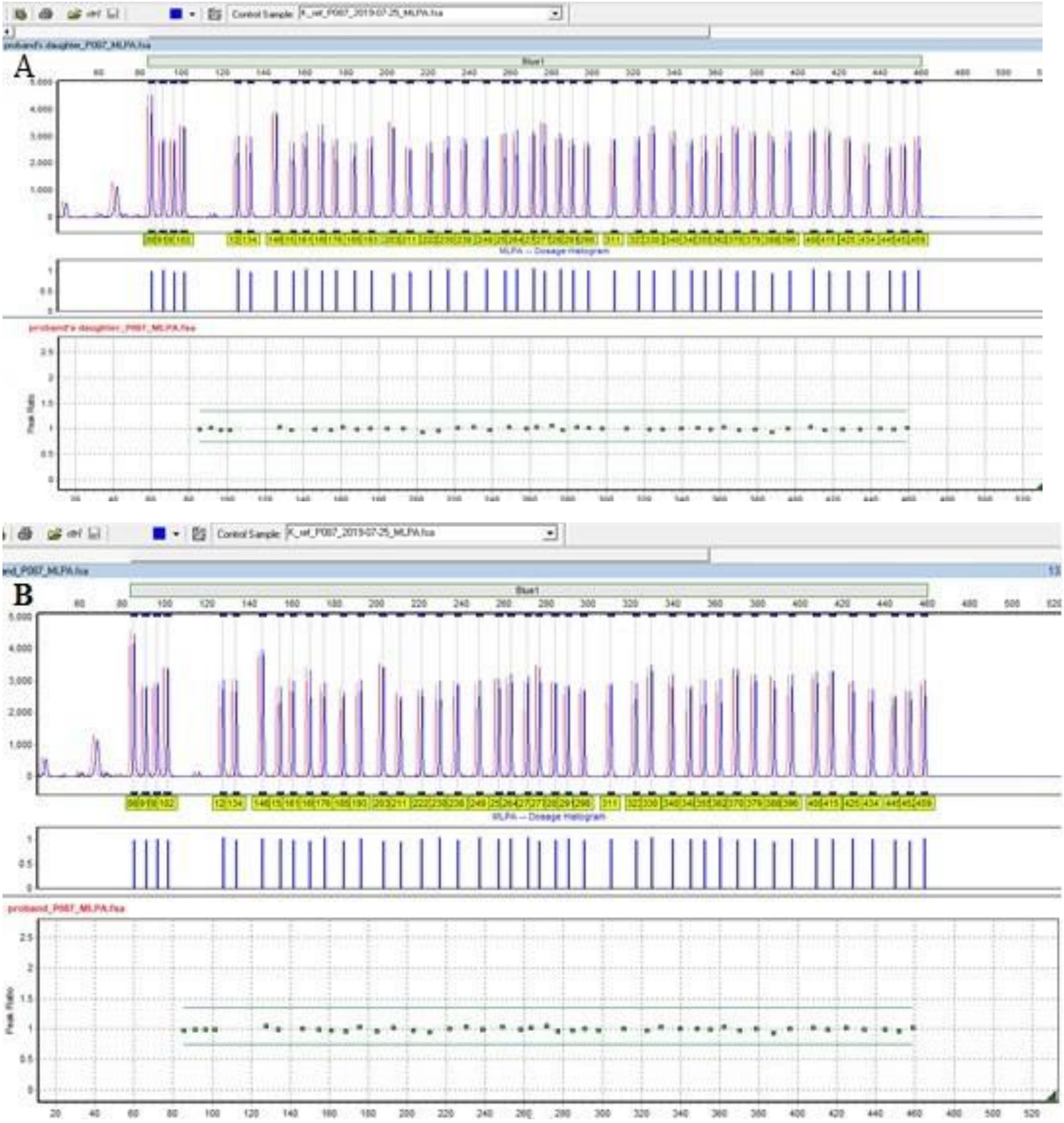

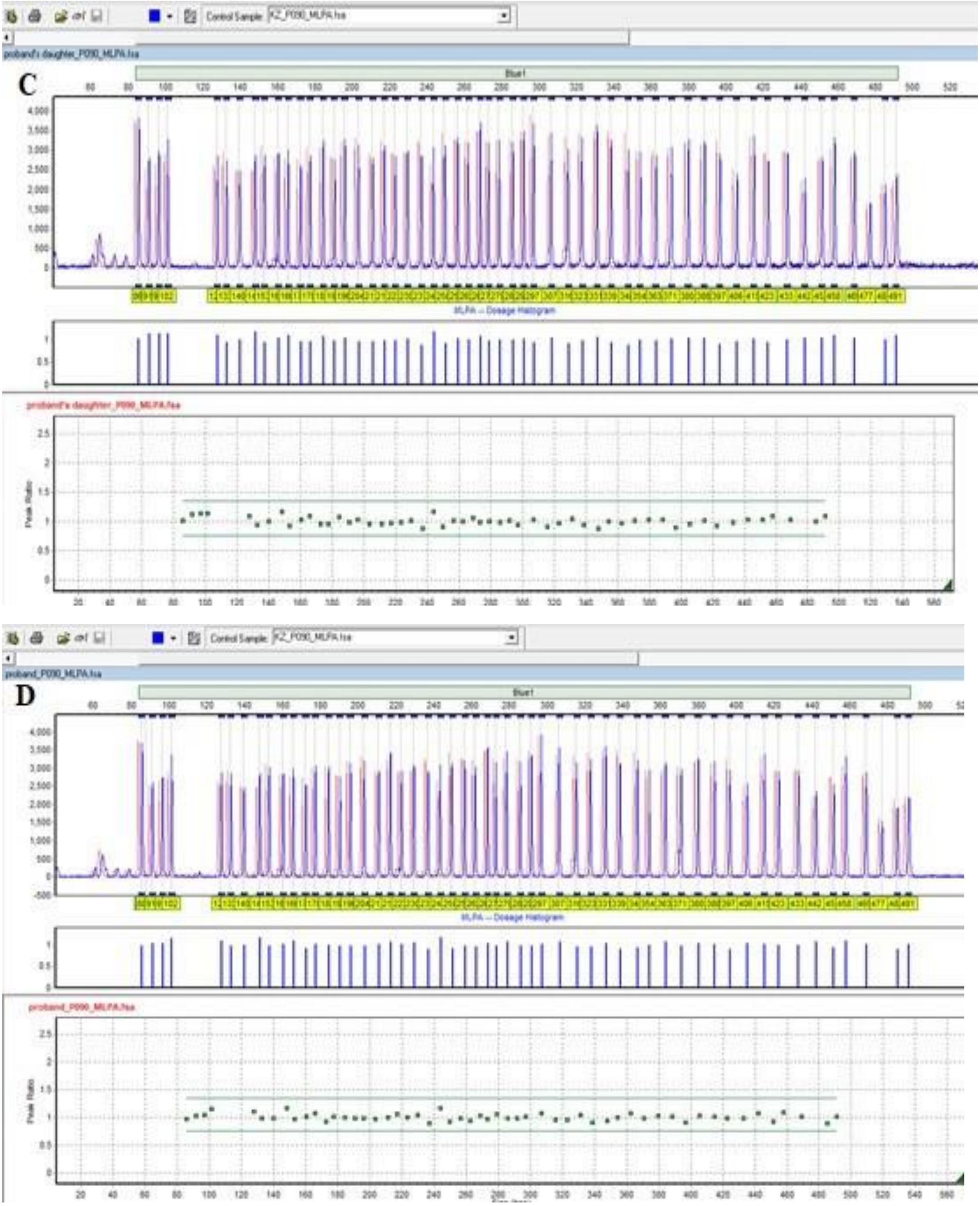

Supplementary figures S1A, S1B, S1C, S1D. *BRCA1/2* genomic arrangements were searched with the MLPA method using P087 SALSA (*BRCA1*) and P090 SALSA MPLA (*BRCA2*) kits. The MLPA analysis demonstrates no deletions or duplications of genomic DNA fragments, i.e., copy number variations (CNV) in proband's daughter (A, C) and proband (B, D) samples. A) P087 proband's daughter, B) P087 proband, C) P090 proband's daughter, D) P090 proband.

Supplementary table S1. Primer sequences used for the analysis of *BRCA1* mutations by Sanger sequencing

**Mutation**	**Primer sequence 5’→3’**	**Annealing temp [˚C]**
c.68_69delAG	F: GGTTGGCAGCAATATGTGAA<br>R: TGTCTTTTCTTCCCTAGTATG	50
c.3700_3704delGTAAA	F: TCCTAGCCCTTTCACCCATACA<br>R: TCCCCAAAAGCATAAACATTT	55
c.3756_3759delGTCT		
c.3779delT		
c.4065_4068delTCAA		
c.4041_4042delAG		
c.829-832delAATA	F: CCTCCAAGGTGTATGAAGTATGT<br>R: TCCAGCCCATCTGTTATGTT	57
